# Mechanism of Formononetin in Improving Energy Metabolism and Alleviating Neuronal Injury in CIRI Based on Nontargeted Metabolomics Research

**DOI:** 10.1111/jcmm.70340

**Published:** 2025-02-24

**Authors:** Jianwen Zhao, Yanwei Zhang, Shuquan Lv, Feng Wang, Ting Shan, Jian Wang, Zeng Liu, Limin Zhang, Huantian Cui, Junbiao Tian

**Affiliations:** ^1^ Hebei Province Cangzhou Hospital of Integrated Traditional and Western Medicine Cangzhou China; ^2^ Hebei Province Key Laboratory of Integrated Traditional and Western Medicine in Neurological Rehabilitation Cangzhou Hospital of Integrated Traditional Chinese Medicine and Western Medicine Cangzhou China; ^3^ Graduate School Chengde Medical University Chengde China; ^4^ Graduate School Hebei University of Chinese Medicine Shijiazhuang China; ^5^ First School of Clinical Medicine Yunnan University of Chinese Medicine Kunming China; ^6^ Hebei Provincial Hospital of Chinese Medicine Shijiazhuang China

**Keywords:** alanine, aspartate and glutamate metabolism, cerebral ischaemia–reperfusion injury, energy metabolism, formononetin, nicotinate and nicotinamide metabolism, oxidative stress, untargeted metabolomics

## Abstract

Cerebral ischaemia–reperfusion injury (CIRI), resulting from thrombolytic therapy for ischaemic stroke, presents a considerable challenge during postoperative recovery. Formononetin (FMN) has shown promise in the prevention and treatment of neurological diseases. However, its specific mechanism in ameliorating CIRI remains uncertain. Initially, we established a CIRI rat model to evaluate FMN's therapeutic potential by assessing neurological function, infarct area and pathological changes. Subsequently, we employed metabolomics technology to investigate FMN's impact on metabolite levels in the ischaemic brain tissue of CIRI rats. Based on the metabolomics findings, we validated FMN's effects on nicotinate and nicotinamide metabolism, as well as alanine, aspartate and glutamate metabolism, along with its influence on neuronal injury and repair. Our investigation unveiled that FMN intervention significantly diminished the Longa score and asymmetry score in CIRI rats, constricted the infarct area and ameliorated pathological alterations in the ischaemic brain tissue, including reduced DCI index and augmented Nissl body count. Metabolomics analysis indicated that FMN exerted regulatory effects on nicotinate and nicotinamide metabolism, as well as alanine, aspartate and glutamate metabolism. Following FMN intervention, there was a notable increase in the levels of related metabolites such as nicotinamide (NAM), L‐aspartic acid (L‐Asp), fumaric acid (FA), gamma‐aminobutyric acid (GABA) and L‐glutamic acid (L‐Glu). RT‐qPCR and Western blot outcomes demonstrated that FMN upregulated the gene and protein expression of key enzymes adenylosuccinate lyase (ADSL) and glutamic acid decarboxylase (GAD) involved in alanine, aspartate and glutamate metabolism. Moreover, FMN intervention bolstered SOD activity, diminished MDA and ROS levels and reduced TUNEL‐positive expression. Furthermore, FMN intervention elevated ATP levels and markedly increased Ki67‐positive expression. FMN exhibits the potential to alleviate oxidative stress injury in CIRI rats by enhancing nicotinate and nicotinamide metabolism along with alanine, aspartate and glutamate metabolism, consequently reinstating energy metabolism and conferring neuroprotective effects to ameliorate CIRI.

**Abbreviations**
ADSL: adenylosuccinate lyase
ATP: adenosine triphosphate
cDNA: complementary DNA
CIRI: cerebral ischaemia–reperfusion injury
DCI: degenerative cell index
FA: fumaric acid
FMN: formononetin
GABA: gamma‐aminobutyric acid
GAD: glutamic acid decarboxylase
GNT: ginaton
HE: haematoxylin and eosin
L‐Asp: L‐aspartic acid
L‐Glu: L‐glutamic acid
MCAO/R: middle cerebral artery occlusion reperfusion
MDA: malondialdehyde
NAM: nicotinamide
PCA: principal component analysis
PLS‐DA: partial least squares‐discriminant analysis
QC: quality control
ROS: reactive oxygen species
SOD: superoxide dismutase
TTC: 2,3,5‐triphenyl tetrazolium chloride
TUNEL: terminal deoxynucleotidyl transferase dUTP nick end labelling

## 1 Introduction

Ischaemic stroke, characterised by its high morbidity, recurrence rate and mortality, presents a significant and challenging medical condition. Clinically, drug thrombolysis serves as the primary approach to swiftly restore blood supply to ischaemic brain tissue. However, the rapid restoration of blood flow can trigger compensatory mechanisms that worsen brain tissue damage, leading to cerebral ischaemia–reperfusion injury (CIRI), profoundly impacting patients' quality of life [1, 2]. Despite considerable advancements in the diagnosis and treatment of CIRI in modern medicine, challenges such as postoperative rehabilitation difficulties and adverse drug reactions persist [3, 4]. Hence, there exists an urgent necessity to explore and develop novel treatment modalities and medications to more effectively tackle the challenges in CIRI management.

The distinctive benefits of natural products in ameliorating CIRI have garnered widespread attention [5]. A randomised controlled clinical study demonstrated that saffron extract effectively enhances the antioxidant capacity of ischaemic stroke patients following thrombolysis, thereby reducing neurological deficits [6]. Another clinical investigation revealed that ginkgolide B regulates brain energy metabolism and tissue oxygenation, consequently improving brain injury [7]. Furthermore, a meta‐analysis highlighted the neuroprotective potential of curcumin in CIRI, attributed to its antioxidant and anti‐inflammatory properties [8]. Understanding the mechanisms underlying the efficacy of natural products in alleviating CIRI holds significant importance for their broad application.

Energy metabolism dysfunction and oxidative stress injury play pivotal roles in exacerbating CIRI [9]. Following cerebral ischaemia, local brain tissue experiences a blockade in oxygen and glucose supply, depleting the energy metabolite adenosine triphosphate (ATP) and triggering an escalation in the ischaemic injury cascade. Upon restoration of blood supply to ischaemic brain tissue, various pathological processes, including oxidative stress and inflammatory reactions, exacerbate mitochondrial damage, thereby worsening energy metabolism dysfunction [10] Studies have validated that reinstating ATP synthesis can effectively enhance neuronal viability and facilitate recovery from CIRI [11]. Furthermore, a meta‐analysis has underscored the significant elevation of oxidative stress levels in stroke patients, with antioxidant therapy demonstrating substantial efficacy in reducing the infarcted brain area and mitigating poor outcomes [12]. Enhancing energy metabolism to bolster neuronal repair stands out as a crucial mechanism in the treatment of CIRI utilising natural products. Ginsenosides have shown efficacy in regulating energy metabolism in CIRI rats, enhancing mitochondrial activity and stimulating ATP production, thereby exerting neuroprotective effects [13].

Formononetin, a prominent member of the isoflavone family, is commonly found in traditional Chinese medicine, such as *Astragalus membranaceus* (Fisch.) Bge. It exhibits pharmacological properties including antioxidation, anti‐infection, anti‐apoptosis and enhanced blood circulation, holding significant promise in the prevention and treatment of neurological disorders like stroke [14, 15]. However, the therapeutic effects and mechanisms of FMN on CIRI remain underexplored. Here, we aim to elucidate the therapeutic potential of FMN on CIRI and further investigate its underlying mechanisms using metabolomics techniques. Initially, we induced a CIRI rat model via middle cerebral artery occlusion reperfusion (MCAO/R) to assess the beneficial effects of FMN on CIRI. Subsequently, metabolomics techniques were employed to explore the impact of FMN intervention on brain tissue metabolites in CIRI rats. Based on the metabolomics results, our focus centred on validating FMN's effects on nicotinate and nicotinamide metabolism, alanine, aspartate and glutamate metabolism, as well as its influence on neuronal injury and repair.

## 2 Methods

### 2.1 Animals and Reagents

We procured SPF‐grade healthy male SD rats, aged 6–8 weeks and weighing around 230 g, from Beijing SPF Biotechnology Co. Ltd., bearing the animal licence number SCXK (Beijing) 2019–0010. Each cage accommodated five rats, and they were maintained under standard SPF‐grade conditions with regular feeding. Approval for all animal experiments was obtained from the Ethical Review Committee of Animal Experiments in Yunnan University of Chinese Medicine (Approval No.: R‐062023LH265), dated March 07, 2023. Details regarding the materials and kits utilised in this experiment are provided in the supporting materials.

### 2.2 Modelling, Grouping and Administration

We established a rat model of cerebral ischaemia–reperfusion injury using the suture occlusion method [16]. Initially, rats underwent an 8‐h fasting period with access to water ad libitum before modelling. Subsequently, they were intraperitoneally anaesthetised with pentobarbital sodium at a dose of 50 mg/kg. Following anaesthesia, rats were positioned supinely, securely fixed and the surgical area was disinfected. A midline incision was made in the neck to expose the left common carotid artery, external carotid artery and internal carotid artery. The external carotid artery was ligated at its bifurcation with the internal carotid artery. A small incision was then made in the left common carotid artery, and a 0.26‐mm‐diameter nylon suture was inserted and utilised to occlude the common carotid artery. The incision was sutured, leaving the suture ends protruding outside the body. Reperfusion was initiated 2 h postsurgery by withdrawing the suture to the level of the common carotid artery. The Sham‐operated group underwent identical surgical procedures, excluding ligation and suture insertion.

We collected tissue samples and measured relevant indicators following a 5‐day reperfusion period. Ninety SD rats were randomly allocated into six groups: Sham‐operated group (Sham), CIRI group, Ginaton group (GNT), low‐dose FMN group (L‐FMN), medium‐dose FMN group (M‐FMN) and high‐dose FMN group (H‐FMN). CIRI models were induced in all groups except the Sham group. Postmodelling, the Sham and CIRI groups received 0.01 mL/g of physiological saline via gavage, while the GNT group was administered 21.6 mg/kg of Ginaton via the same route. The L‐FMN, M‐FMN and H‐FMN groups were orally administered 15, 30 and 60 mg/kg of FMN, respectively, once daily for five consecutive days. The dosage of GNT and FMN was set according to previous studies [17, 18].

**FIGURE 1:**
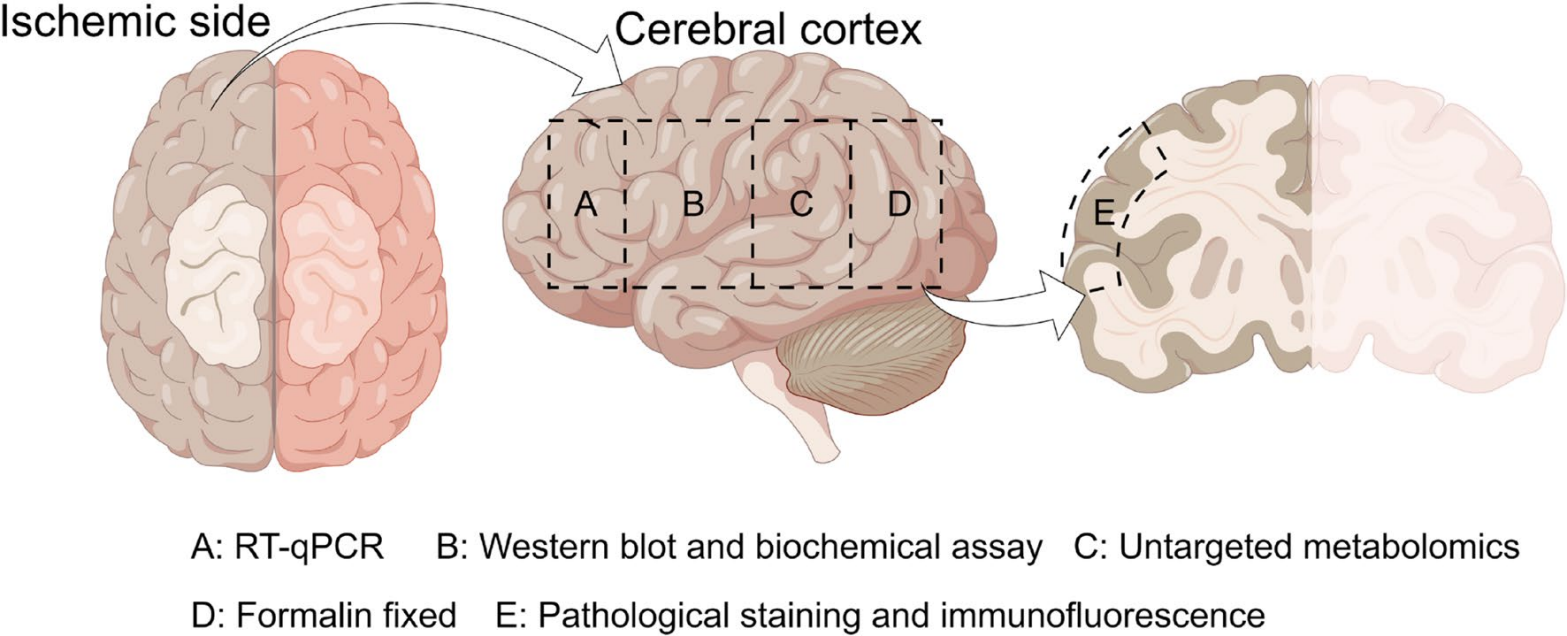
Rat brain atlas for experimental assay.

After model establishment and drug administration, six rats from each group were used for 2,3,5‐triphenyl tetrazolium chloride (TTC) staining, while the cerebral cortex tissue from the ischaemic side of the brains from the remaining nine rats was divided into four parts for further experiments, including pathological staining, untargeted metabolomics analysis, detection of SOD, MDA, ROS and ATP in brain tissue, RT‐qPCR, Western blot and immunofluorescence assays (Figure 1).

### 2.3 Neurological Function Evaluation

We utilised the Longa score [19] and asymmetry score [20] to evaluate neurological dysfunction in rats. The Longa score, a scale ranging from 0 to 4, delineates the severity of deficits: 0 signifies no observable neurological impairment, 1 denotes an inability to extend the contralateral forepaw, 2 signifies circling towards the contralateral side, 3 indicates falling towards the contralateral side and 4 represents loss of consciousness and spontaneous ambulation. Meanwhile, the asymmetry score, determined by the frequency of forelimb contact with the cage when the tail is elevated, complements this assessment. The calculation formula is as follows:

$$\text{Asymmetry score}=\frac{\text{Left}-\text{Right}}{\text{Both}+\text{Left}+\text{Right}}\times 100\%$$

### 2.4 Cerebral Infarction Area Assessment

We evaluated the infarct area in CIRI rats based on prior research [21]. In this procedure, six rats were randomly chosen from each group, anaesthetised and their brain tissues were dissected and subsequently stored in a − 20°C refrigerator for 30 min. Subsequently, the brains were embedded in a brain‐slicing mould and sliced coronally into 2‐mm‐thick sections. These sections were then immersed in a 2% TTC staining solution and incubated in darkness at 37°C for 30 min, with rotation every 5 min to ensure consistent staining. Following staining, the brain sections underwent photography and documentation. The infarct area in each rat group was precisely quantified using Image J software.

### 2.5 Pathological Staining

These sections underwent staining with haematoxylin and eosin (HE) [22], Nissl [23] and terminal deoxynucleotidyl transferase dUTP nick end labelling (TUNEL) [24] staining methods, as per established protocols. Briefly, cerebral cortex tissue from the same ischaemic region was obtained, fixed with 4% paraformaldehyde, dehydrated with alcohol and embedded in paraffin. The tissue was then cut into 5‐μm sections, which were dewaxed with xylene and washed with water for 20 min. Subsequently, the sections were stained with haematoxylin and eosin or Nissl stain using 0.1% toluidine blue. For TUNEL staining, 2‐μm sections were prepared and stained following the instructions provided in the TUNEL kit. Subsequently, we observed the sections under a microscope. The extent of pathological damage in the HE‐stained sections was evaluated using the degenerative cell index (DCI), computed as the ratio of degenerative cells to the total cell count [25]. Quantitative analysis of Nissl bodies and TUNEL‐positive areas was conducted using Image J software.

### 2.6 Untargeted Metabolomics Analysis

We collected brain tissue samples from the ischaemic side, which were then ground using liquid nitrogen and diluted with water at a mass‐to‐volume ratio of 1:3 to create a tissue suspension. Subsequently, we added methanol containing internal standards at a volume ratio of 1:4, mixed the solution, allowed it to stand, centrifuged it and transferred the supernatant to GC vials. The supernatant was then transformed into a dry powder using a concentration instrument. This dry powder was combined with a methoxyamine pyridine solution, allowed to stand for a defined period and then mixed with N‐methyl‐N‐(trimethylsilyl) trifluoroacetamide, followed by another period of standing. Finally, we added an external standard solution, mixed it and analysed it using the instrument. We prepared quality control (QC) samples by mixing equal volumes of each sample. Further details on sample processing, GC/MS detection and data analysis are available in the supporting materials.

### 2.7 SOD, MDA, ROS, ATP Detection in Brain Tissue

We collected cerebral cortex tissue from the ischaemic side and homogenised the tissue. We normalised the total protein concentration of the samples using the BCA method. Following this, we detected the activity of SOD and measured the levels of MDA, ROS and ATP in the brain tissue, adhering to the instructions provided in the kit.

### 2.8 RT‐qPCR Detection

Following the extraction of total RNA from cerebral cortex tissue of the ischaemic side, we added TRIzol reagent to preserve RNA integrity. Subsequently, we measured the purity and concentration of total RNA and performed reverse transcription to generate complementary DNA (cDNA). The samples were then loaded into a 96‐well plate for PCR amplification. We recorded the cycle threshold (C_t_ value) for each reaction tube, indicating the number of cycles required for the fluorescent signal to reach a predefined threshold. Utilising the 2^−ΔΔC^_t_ method, we calculated the relative expression levels of each target mRNA compared to *Actb*. Primer sequences are available in the (Table S1).

### 2.9 Western Blot Assay

We retrieved cerebral cortex tissue of the ischaemic side from the −80°C freezer, minced it and placed it in a 1.5‐mL EP tube. RIPA lysis buffer was then added, and the tissue underwent homogenisation and lysis for 30 min. Following centrifugation, we collected the supernatant and determined the concentration of total protein using the BCA method. The protein was subsequently separated via SDS‐PAGE electrophoresis and transferred to a PVDF membrane. After blocking the membrane with 5% skim milk and washing it with TBST, we added the primary antibody against the target protein, allowing it to incubate overnight at 4°C. The next day, we washed the membrane five times with TBST and applied the appropriate secondary antibody for a 2‐h incubation on a rocker at 37°C. Following another round of washing with TBST, we developed the membrane using ECL and exposed it. Analysis was conducted using Image J software, with β‐actin serving as an internal control for the calculation of relative expression levels of the target protein.

### 2.10 Immunofluorescence Assay

We processed the fixed cerebral cortex tissue of the ischaemic side through gradient ethanol dehydration, clearing, wax immersion and embedding for slicing. After dewaxing the sections with xylene and ethanol, we performed antigen retrieval and blocked them with goat serum for 30 min. Ki67 antibodies were then applied and left to incubate overnight in a dark, humid chamber at 4°C. Following this, we added fluorescent labels and allowed them to incubate for 1 h at 37°C in a humid chamber. Nuclei were stained with DAPI in a dark setting, and the slides were mounted with antifade mounting medium for observation. We quantified the positive regions using Image Pro Plus 6.0 software.

### 2.11 Statistical Analysis

We conducted statistical analysis using SPSS Pro, and we presented all data as mean ± SD. When data adhered to a normal distribution and exhibited homogeneity of variance among groups, we employed a *t*‐test or one‐way ANOVA. Alternatively, if the data did not meet the criteria for normal distribution, we utilised a rank‐sum test for analysis. We considered a *p*‐value less than 0.05 as statistically significant.

## 3 Results

### 3.1 The Therapeutic Effect of FMN on CIRI Rats

**FIGURE 2:**
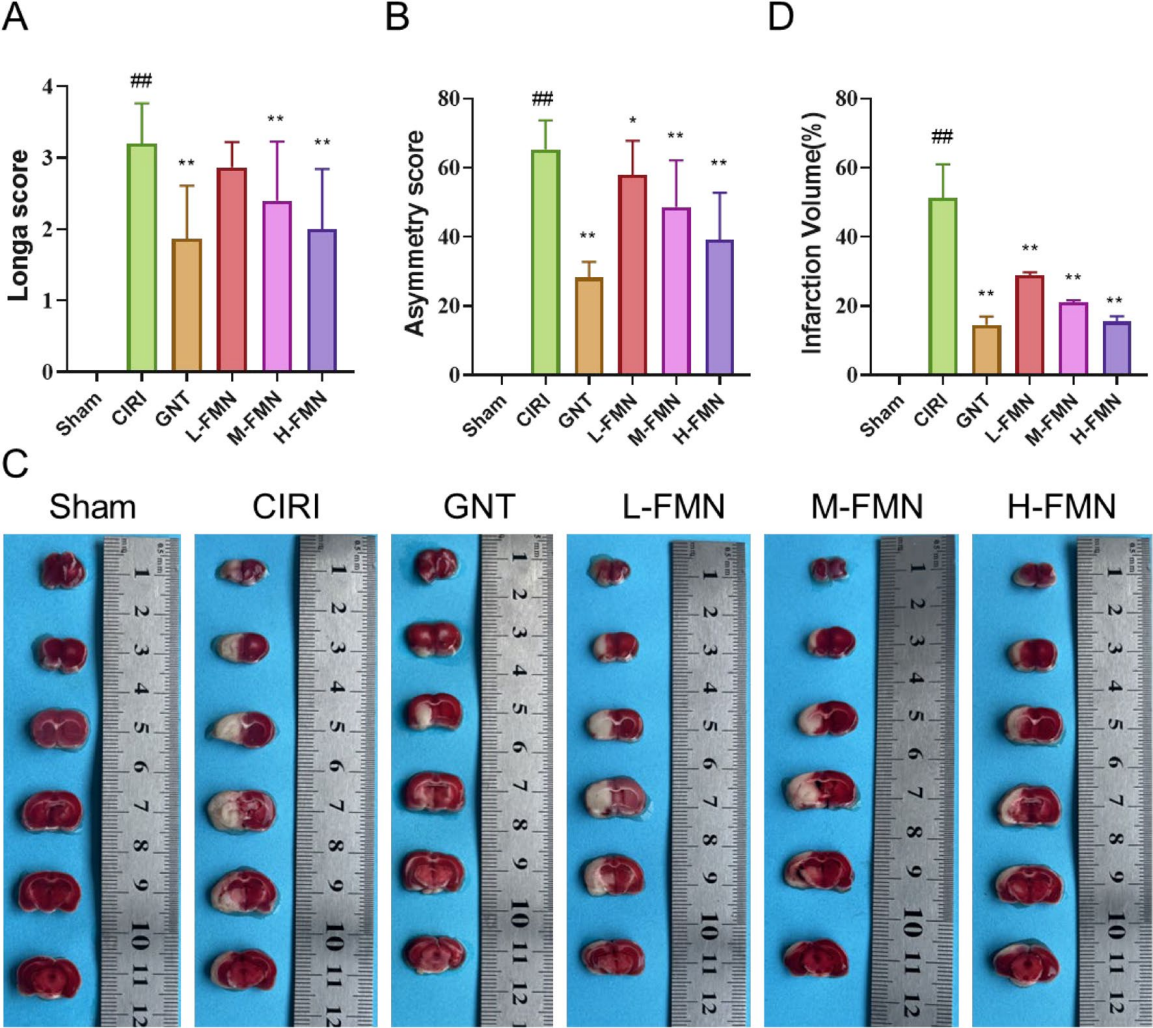
FMN intervention can restore nerve function and shrink infarct areas of CIRI rats. After establishing the CIRI model in rats through MCAO/R, we administered FMN at doses of 15, 30 and 60 mg/kg. We subsequently evaluated the neurological damage in the rats using the Longa score and the asymmetry score, as well as assessed cerebral infarction in the rats through TTC staining. (A, B) FMN intervention significantly reduces the Longa score (A) and asymmetry score (B) in CIRI rats. (C, D) TTC staining demonstrates that the FMN intervention significantly reduces the area of cerebral infarction in CIRI rats. Data are presented as mean ± SD. *n* = 15 per group for A, B; *n* = 6 per group for C, D. ##*p* < 0.01 versus Sham group; *p < 0.05, **p < 0.01 versus CIRI group. CIRI, cerebral ischaemia– reperfusion injury; FMN, formononetin; GNT, ginaton; MCAO/R, middle cerebral artery occlusion reperfusion; TTC, 2,3,5‐triphenyl tetrazolium chloride.

**FIGURE 3:**
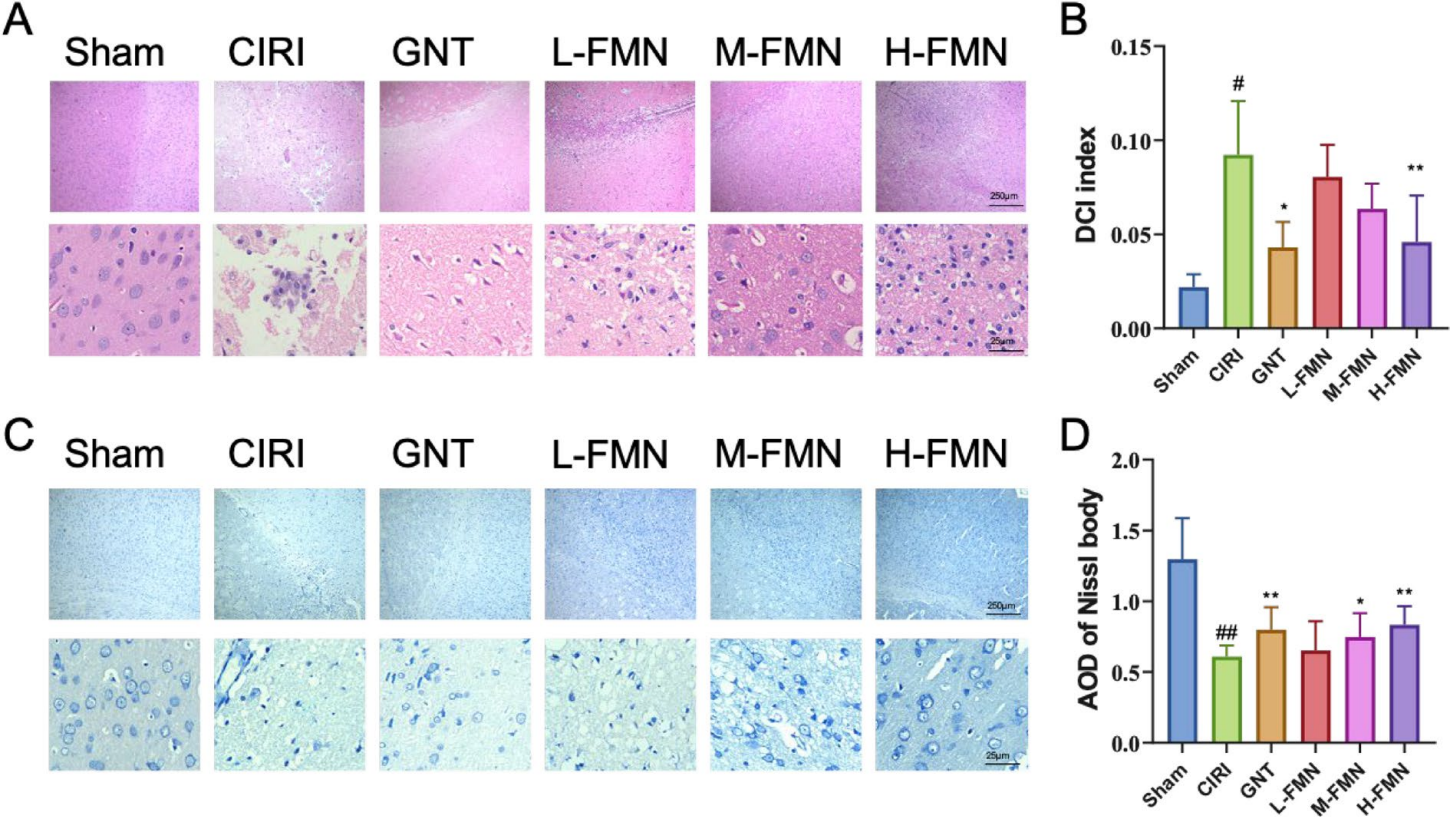
FMN intervention effectively improves pathological injury of brain tissue in CIRI rats. We utilised HE staining and Nissl staining to evaluate the effects of FMN on pathological changes in the ischaemic regions of cerebral cortex tissue in CIRI rats. (A, B) HE staining reveals that FMN intervention improves the pathological state and DCI index of brain tissue in CIRI rats. (C, D) Nissl staining shows that FMN intervention increases the mean optical density of Nissl bodies in brain tissue. The magnification for both HE staining and Nissl staining is 40× and 400×. *n* = 9 per group. #*p* < 0.05, versus Sham group; *p < 0.05, **p < 0.01 versus CIRI group. DCI, degenerative cell index; HE, haematoxylinhematoxylin and eosin; #*P* < 0.05, ##*P* < 0.01 versus Sham group.

Throughout the evaluation of FMN's therapeutic effect on CIRI rats, we utilised the Longa score and asymmetry score to gauge neurological dysfunction (Figure 2A,B). We employed TTC staining to assess cerebral infarction and HE along with Nissl staining to evaluate brain tissue pathology. Our findings from the Longa score and asymmetry score indicated that CIRI rats displayed notably higher scores compared to the Sham group, signifying neurological impairment. However, intervention with FMN significantly mitigated these deficits in CIRI rats. TTC staining illustrated a significant increase in the infarct area in CIRI rats compared to the Sham group, yet after 5 days of FMN intervention, this area notably decreased (Figure 1C,D). HE staining unveiled pathological changes in the brain tissue of CIRI rats, including increased glial cells, loose intercellular substance and nuclear pyknosis, in contrast to the Sham group. FMN intervention effectively attenuated these damages (Figure 3A,B). Nissl staining demonstrated a significant reduction in the number of Nissl bodies in CIRI rats compared to the Sham group, which was reversed by FMN intervention (Figure 3C,D). The therapeutic efficacy of different doses of FMN suggested a dose‐dependent response in treating CIRI. Consequently, we proceeded with further analysis focusing on the H‐FMN group.

### 3.2 The Effects of FMN on the Metabolic Pathways in the Brain Tissue of CIRI Rats

**FIGURE 4:**
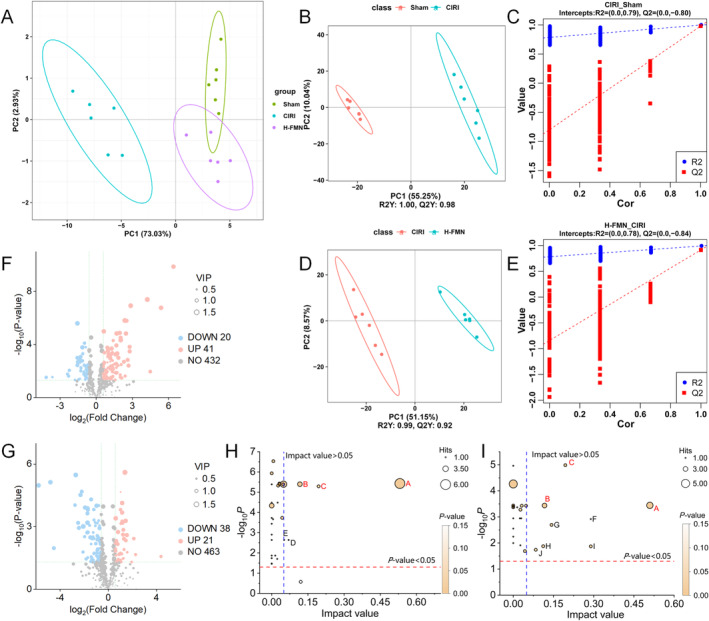
Effect of FMN intervention on metabolic pathways in brain tissue of CIRI rats evaluated based on untargeted metabolomics. (A) PCA reveals significant differences in metabolic characteristics between the CIRI and Sham groups, as well as between the H‐FMN and CIRI groups. (B–E) PLS‐DA score plots and permutation tests between (B, C) the CIRI and Sham groups, and (D, E) the H‐FMN and CIRI groups. The statistical models exhibited good fitness and predictive ability. (F, G) Differential metabolites in (F) CIRI versus Sham, and (G) H‐FMN versus CIRI. (H, I) Pathway analysis results for (H) the CIRI and Sham groups, and (I) the H‐FMN and CIRI groups. Nicotinate and nicotinamide metabolism, alanine, aspartate and glutamate metabolism, and arginine biosynthesis were the intersecting pathways as marked in red. All significantly different pathway names are represented by ‘A–J’: A, Alanine, aspartate and glutamate metabolism, B, Arginine biosynthesis, C, Nicotinate and nicotinamide metabolism, D, Glycolysis/Gluconeogenesis, E, Fructose and mannose metabolism, F, Arachidonic acid metabolism, G, Phenylalanine metabolism, H, Pentose phosphate pathway, I, Pentose and glucuronate interconversions, J, Sphingolipid metabolism. *n* = 6 per group. PCA, principal component analysis; PLS‐DA, partial least squares‐discriminant analysis.

Principal component analysis (PCA) of nontargeted metabolomics in rat brain tissue samples delineated clear separations among the Sham, CIRI and H‐FMN groups, with clustered data within each group (Figure 4A). This observation indicates notable differences in metabolite levels within brain tissue among the three groups. Utilising the PLS‐DA model to identify differential metabolites and validating the model via permutation testing, we obtained *R* ^2^ values of (0.00, 0.79) and *Q* ^2^ values of (0.00, −0.80) for CIRI versus Sham, and *R* ^2^ values of (0.00, 0.78) and *Q* ^2^ values of (0.00, −0.84) for H‐FMN versus CIRI. These findings signify the robust fitting and predictive capacity of the statistical model (Figure 4B–E).

Subsequently, we screened the differential metabolites among the groups based on the criteria of *p* < 0.05, VIP > 1, and fold change greater than 1.5 or less than 0.67. And differential metabolites were visualised by volcano plots (Figure 4F,G). Comprehensive information on the identified differential metabolites is available in the supporting materials (differential metabolites of CIRI vs. Sham are shown in Table S2 and differential metabolites of H‐FMN vs. CIRI groups are shown in Table S3). We conducted KEGG pathway enrichment analysis on the identified differential metabolites using MetaboAnalyst 6.0. The selection criteria for key pathways were *p* < 0.05 and pathway impact > 0.05. ‘Impact’ is used to evaluate the importance and contribution of a metabolic pathway. Additionally, ‘Hits’ reflect the number of metabolites detected within that pathway. The outcomes revealed nicotinate and nicotinamide metabolism (map00760) and alanine, aspartate and glutamate metabolism (map00250) as key pathways for CIRI versus Sham, and H‐FMN versus CIRI. Notably, these pathways overlapped, suggesting their significance as key metabolic pathways for FMN‐mediated improvement in CIRI (Figure 4H,I). Hence, we proceeded to validate the key factors within these pathways.

### 3.3 Effects of FMN on Antioxidative‐Related Pathway and Oxidative Stress Injury in Brain Tissue

**FIGURE 5:**
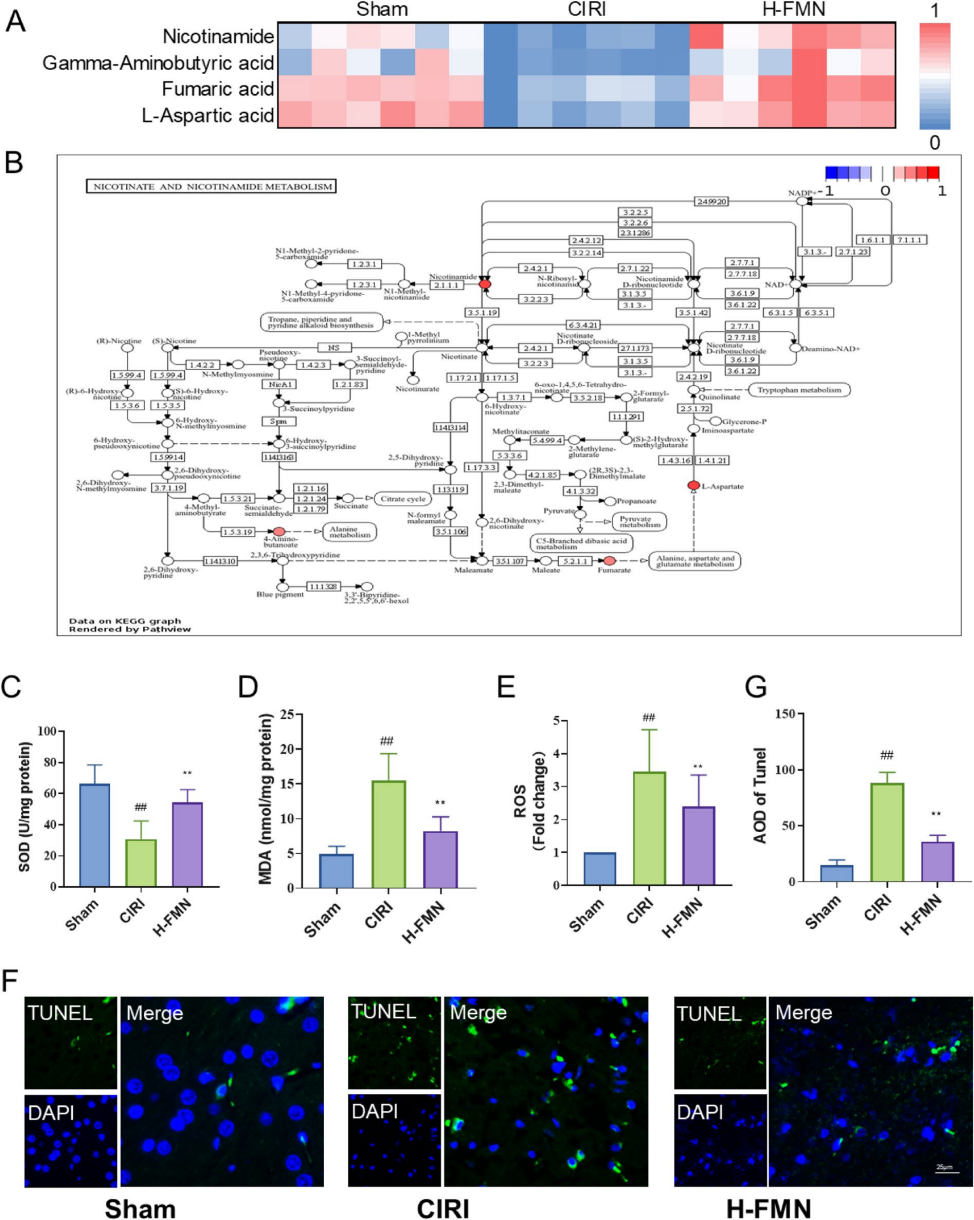
FMN regulates nicotinate and nicotinamide metabolism and inhibits oxidative stress. (A) FMN upregulates levels of metabolites related to nicotinate and nicotinamide metabolism, including NAM, L‐Asp, FA and GABA. (B) Pathway map reveals that NAM is the core metabolite of nicotinate and nicotinamide metabolism, while L‐Asp, FA, and GABA all contribute to Alanine, aspartate, and glutamate metabolism. The colour bar represents the relative expression levels of the metabolites of H‐FMN versus CIRI. (C–E) Antioxidant experiments demonstrate that FMN can enhance SOD activity (C), as well as reduce the levels of MDA (D) and ROS (E) in brain tissue. (F, G) TUNEL staining shows that FMN intervention reduces cell death caused by oxidative stress. *n* = 6 per group for A, B. *n* = 9 per group for C–G. ##*p* < 0.01 versus Sham group; **p < 0.01 versus CIRI group. FA, fumaric acid; GABA, gamma‐aminobutyric acid; L‐Asp, L‐aspartic acid; MDA, malondialdehyde; NAM, nicotinamide; ROS, reactive oxygen species; SOD, superoxide dismutase; TUNEL, terminal deoxynucleotidyl transferase dUTP nick end labelinglabelling.

In nicotinate and nicotinamide metabolism, our screening process identified nicotinamide (NAM), L‐aspartic acid (L‐Asp), fumaric acid (FA) and gamma‐aminobutyric acid (GABA) as key metabolites, which were consistent with previous reports [26]. Relative to the Sham group, these metabolite levels significantly decreased in the CIRI group, whereas FMN intervention elevated their levels (Figure 5A). Examination of the distribution and expression of these metabolites in nicotinate and nicotinamide metabolism revealed that L‐Asp, FA and GABA serve as upstream or downstream products of alanine, aspartate and glutamate metabolism, thereby corroborating FMN's role in enhancing this metabolic pathway (Figure 5B). NAM, a central metabolite in nicotinate and nicotinamide metabolism, is well‐known for its antioxidant properties [26]. Given FMN's ability to increase NAM levels, we assessed FMN's antioxidant effects in CIRI. Our findings demonstrated that FMN significantly boosted SOD activity in ischaemic brain tissue and lowered MDA and ROS levels (Figure 5C–E). Furthermore, to evaluate FMN's efficacy in mitigating cell damage induced by oxidative stress, we performed TUNEL staining, which revealed FMN's substantial amelioration of cellular damage caused by oxidative stress (Figure 5F,G).

### 3.4 Effects of FMN on Key Energy Metabolism and Brain Tissue Repair

**FIGURE 6:**
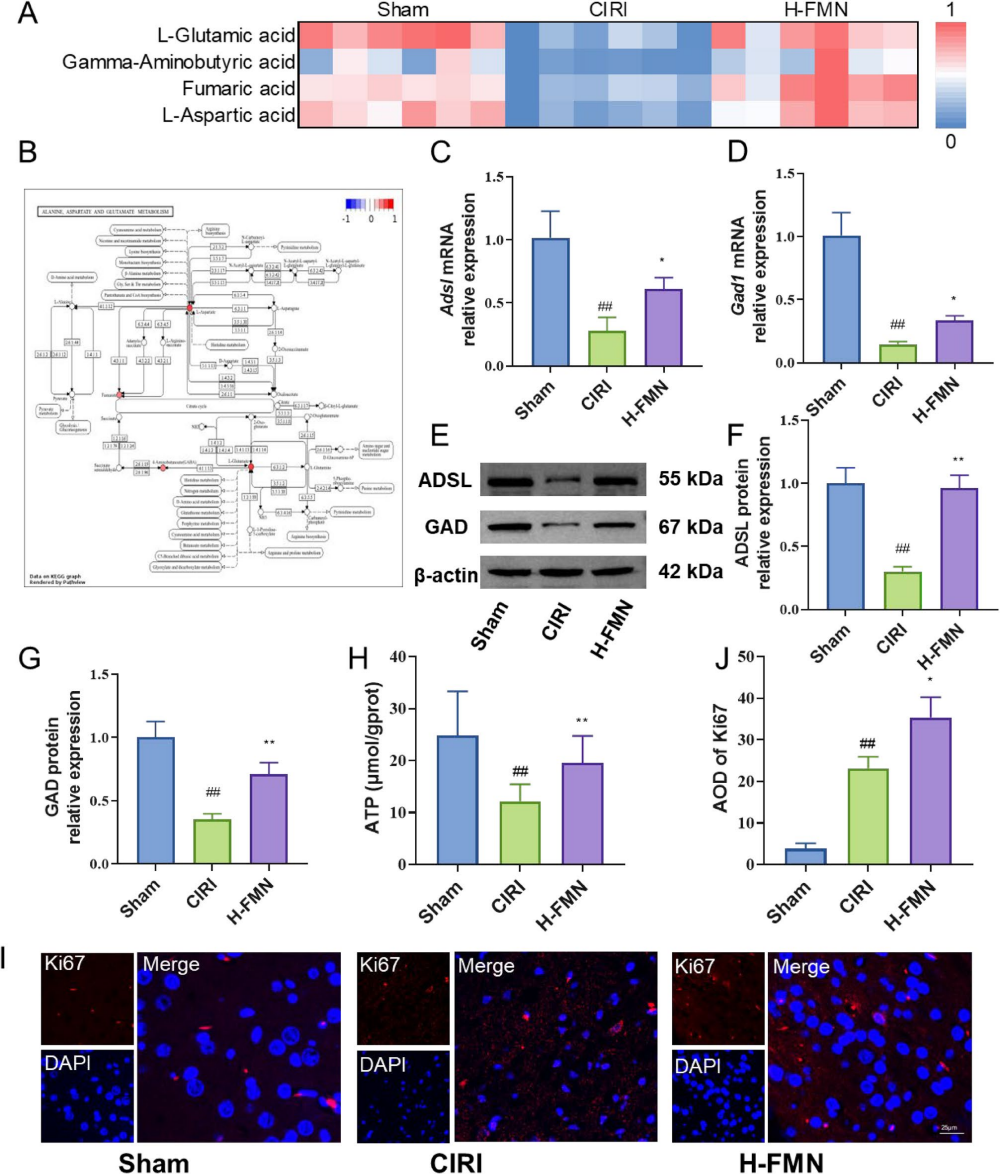
FMN regulates key energy metabolism and induces neuronal proliferation. (A) FMN upregulates the levels of metabolites related to alanine, aspartate and glutamate metabolism, including L‐Asp, FA, GABA and L‐Glu. (B) Pathway map indicates that L‐Asp, FA, GABA and L‐Glu are upstream products of the TCA cycle, and ADSL and GAD are key enzymes involved in the metabolism of these metabolites. (C–G) RT‐qPCR and Western blot results show that FMN intervention upregulates gene and protein expression of ADSL (C, E, F) and GAD (D, E, G). (H) FMN intervention upregulates the level of ATP. (I, J) Immunofluorescence results demonstrate that FMN intervention increases the positive expression of Ki67. *n* = 6 per group for A–G. *n* = 9 per group for H–J. ##*p* < 0.01 versus Sham group; *p < 0.05, **p < 0.01 versus CIRI group. ADSL, adenylosuccinate lyase; ATP, adenosine triphosphate; GAD, glutamic acid decarboxylase; L‐Glu, L‐glutamic acid; TCA, tricarboxylic acid.

Building upon our preceding research, we directed our focus towards validating alanine, aspartate and glutamate metabolism. Specifically, L‐Asp, FA, GABA and L‐glutamic acid (L‐Glu) emerged as key metabolites in our screenings. Our findings unveiled a significant reversal in the reduction of these metabolites following FMN intervention (Figure 6A). Examination of the distribution and expression of these metabolites within alanine, aspartate and glutamate metabolism highlighted their role as upstream products of the tricarboxylic acid (TCA) cycle, thereby influencing energy metabolism in brain tissue. adenylosuccinate lyase (ADSL) and glutamic acid decarboxylase (GAD) emerged as pivotal enzymes governing the metabolism of these metabolites (Figure 6B). Consequently, we proceeded to assess the impact of FMN on the gene and protein expression of ADSL and GAD through RT‐qPCR and Western blot techniques. Our results demonstrated that FMN notably upregulated the gene and protein expression of ADSL and GAD (Figure 6C–G). Furthermore, we evaluated changes in ATP levels in rat brain tissue, revealing that FMN intervention bolstered ATP content in the ischaemic brain tissue of CIRI rats, thereby enhancing their energy metabolism and exerting neurotrophic effects (Figure 6H). This assertion was corroborated by Ki67 immunofluorescence results, which illustrated FMN's significant promotion of neuronal cell proliferation (Figure 6I,J).

## 4 Discussion

CIRI poses a considerable challenge for the outcomes of ischaemic stroke [27]. In this study, we assessed neural functional impairment in rats utilising the Longa score [19] and asymmetry score [20]. The Longa score focuses on evaluating neurological deficits and recovery, while the asymmetry score emphasises motor asymmetry assessment. Our findings indicate that FMN effectively mitigates neurological deficits in CIRI rats. Previous research has highlighted the cascade reaction during cerebral ischaemia–reperfusion, exacerbating brain injury and resulting in enlarged infarct areas, increased cellular damage, elevated glial cell counts and reduced neuron numbers [28]. However, our staining results with TTC, HE and Nissl reveal that FMN significantly reverses these pathological conditions in CIRI rats. This reversal encompasses reductions in enlarged infarct areas, decreased glial cell counts and increased Nissl bodies, suggesting FMN's potential as a therapeutic agent for CIRI. Furthermore, our comparison with Ginaton as a positive control [17] demonstrates that high‐dose FMN yields comparable efficacy to Ginaton in CIRI treatment.

Previous studies have shown that the molecular mechanisms by which FMN improves cerebral ischaemia–reperfusion injury (CIRI) include regulating the JAK2/STAT3 signalling pathway [18], the PARP‐1/PARG/Iduna signalling pathway [29] and the PI3K/Akt signalling pathway [30], inhibiting endoplasmic reticulum stress and apoptosis [30, 31] and enhancing cerebrovascular neovascularisation [32]. However, no studies have yet elucidated the metabolic regulatory mechanisms by which FMN improves CIRI. Therefore, we conducted a preliminary exploration of the metabolic mechanism of FMN on CIRI based on untargeted metabolomics. We found that FMN mitigated brain injury and conferred neuroprotective effects by modulating nicotinate and nicotinamide metabolism, as well as alanine, aspartate and glutamate metabolism.

Prior studies have underscored the crucial inhibitory role of nicotinate and nicotinamide metabolism in oxidative stress–induced damage [33]. Our findings indicate that FMN upregulates the levels of NAM, a core metabolite in nicotinate and nicotinamide metabolism. NAM, the amide form of niacin, serves as an essential precursor of nicotinamide adenine dinucleotide and is pivotal for energy metabolism and cellular function. Research has confirmed NAM's ability to inhibit oxidative stress in mouse models of Parkinson's disease, thereby exerting neuroprotective effects [34]. During cerebral ischaemia–reperfusion injury, an imbalance between oxidation and antioxidation within neurons leads to the generation of excessive oxygen free radicals [35]. These radicals attack reperfused neurons and healthy neighbouring neurons, exacerbating neural functional impairment [36]. Supplementation with NAM effectively boosts the body's antioxidant capacity, enhances superoxide dismutase (SOD) activity and reduces levels of malondialdehyde (MDA) and reactive oxygen species (ROS), thus mitigating oxidative stress–induced damage [37]. Our study demonstrates that FMN upregulates NAM levels. Subsequent assessments of oxidative stress factors indicate that FMN intervention enhances the antioxidant capacity of CIRI rats and reduces oxidative stress–induced neuronal damage.

Furthermore, L‐Asp FA, and GABA are vital metabolites in nicotinate and nicotinamide metabolism, serving as crucial upstream and downstream metabolites in alanine, aspartate and glutamate metabolism. Within the alanine, aspartate and glutamate metabolism pathway, L‐Asp, recognised for its antifatigue properties, undergoes metabolisation into FA by ADSL. Previous investigations have highlighted FA's antioxidant attributes, its facilitation of DNA damage repair and its role in mitigating cellular injury. Crucially, FA is integral to energy metabolism within the TCA cycle [38]. Our research findings demonstrate that FMN effectively reverses the decline in L‐Asp and FA levels while upregulating ADSL expression. This suggests FMN's capacity to enhance energy metabolism in CIRI rats by modulating L‐Asp and FA. Notably, our results are substantiated by the significant increase in ATP content observed in ischaemic brain tissue following FMN intervention. Furthermore, the crucial roles of L‐Glu and GABA in neurological diseases have gained widespread recognition, as they collaborate to regulate nervous system function [39]. L‐Glu, serving as a metabolic precursor of GABA, undergoes decarboxylation by GAD to generate GABA. A portion of the L‐Glu and GABA present in the synaptic cleft undergoes further conversion into glutamine (Gln) and L‐Glu through the reuptake mechanism facilitated by glial cells. This metabolic loop involving L‐Glu/GABA‐Gln maintains the balance between L‐Glu‐mediated neural excitation and GABA‐mediated neural inhibition, thereby conferring neuroprotective effects [40]. Moreover, GABA can also participate in the TCA cycle as a precursor of succinate, thereby regulating the body's energy metabolism [41]. Our findings indicate that FMN enhances the levels of L‐Glu and GABA while upregulating GAD expression, suggesting FMN's neuroprotective effects on CIRI rats through modulation of L‐Glu and GABA. This conclusion is further supported by the immunofluorescence results of Ki67.

Of course, the metabolism of an organism is a dynamic process, and the metabolic changes observed in our research represent merely a temporal snapshot. While this may not fully encapsulate the overall metabolic dynamics of disease progression, the metabolic differences within this temporal snapshot offer us a glimpse into significant changes. In future research, the application of spatial metabolomics and metabolic flux technology will facilitate the observation of the holistic dynamic changes of metabolites during disease progression, thereby further elucidating the pharmacological mechanisms of FMN in a comprehensive manner. Furthermore, transcriptomics offers a comprehensive analysis of gene expression profiles in cells under specific conditions, and combining the results of transcriptomics is expected to provide new insights into the molecular mechanisms by which FMN exerts neuroprotective effects. These in‐depth mechanistic studies will facilitate the discovery of specific targets for FMN on CIRI and provide essential basic research data for the development of FMN‐related clinical medications.

## 5 Conclusion

**FIGURE 7:**
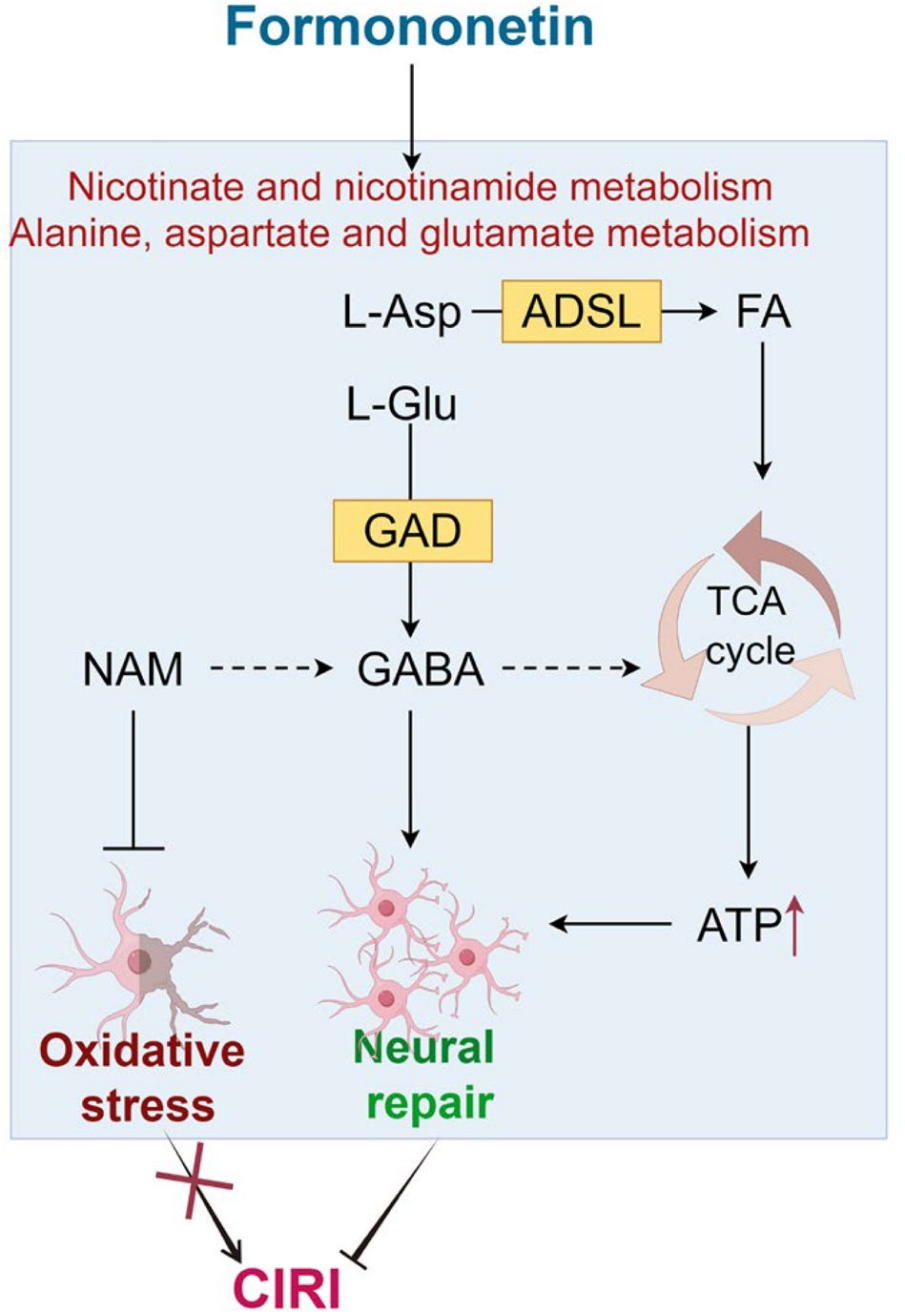
FMN can mitigate oxidative stress injury in CIRI rats and promote the recovery of energy metabolism in CIRI rats by enhancing nicotinate and nicotinamide metabolism, as well as alanine, aspartate and glutamate metabolism, thereby exerting neuroprotective effects.

In summary, FMN exhibits considerable therapeutic promise as a potential treatment for CIRI. Mechanistic studies utilising nontargeted metabolomics indicate that FMN mitigates oxidative stress damage and promotes the restoration of energy metabolism in CIRI rats by modulating nicotinate and nicotinamide metabolism, as well as alanine, aspartate and glutamate metabolism, thus conferring neuroprotective effects (Figure 7). This study provides new insights into the metabolic mechanism of FMN on CIRI and offers candidates for the development of neurorepair drugs for CIRI from the perspective of energy metabolism.

## Author Contributions

**Jianwen Zhao:** funding acquisition (equal), investigation (equal), writing – original draft (equal). **Yanwei Zhang:** data curation (equal), investigation (equal). **Shuquan Lv:** formal analysis (equal), validation (equal). **Feng Wang:** formal analysis (equal), validation (equal). **Ting Shan:** formal analysis (equal), validation (equal). **Jian Wang:** investigation (equal), visualization (equal). **Zeng Liu:** investigation (equal), visualization (equal). **Limin Zhang:** writing – review and editing (equal). **Huantian Cui:** conceptualization (lead). **Junbiao Tian:** conceptualization (equal), writing – review and editing (equal).

## Conflicts of Interest

The authors declare no conflicts of interest.

## Supporting information


Data S1.

## Data Availability

Data will be made available on request. All data were generated in‐house, and no paper mill was used. All authors agreed to be accountable for all aspects of work ensuring integrity and accuracy.
