# Supplementary material for: Mechanism of Formononetin in Improving Energy Metabolism and Alleviating Neuronal Injury in CIRI Based on Nontargeted Metabolomics Research
Source: J Cell Mol Med. 2025 Feb 24;29(4):e70340. doi: 10.1111/jcmm.70340 (PMC11850093; doi:10.1111/jcmm.70340)
Supplement: Supplementary file 1 — Data S1. [file JCMM-29-e70340-s001.docx]

**Supplementary Materials**

**1 Reagents**

Ginaton (H20090365) was purchased from Dr. Willmar Schwabe GmbH & Co. KG (Karlsruhe, Germany). SOD assay kit (A001-3-2), MDA assay kit (A003-1-2), ROS assay kit (E004-1-1), and ATP assay kit (A095-1-1) were purchased from Nanjing Jiancheng Bioengineering Research Institute Co., LTD. Primary antibodies: rabbit anti-ADSL (ab154872, 1:10000), rabbit anti-GAD1 (ab213508, 1:1000), and rabbit anti-Ki67 (ab16667, 1:500) were purchased from Abcam (Shanghai, China). Secondary antibody for goat anti-rabbit IgG H&L (ab207995, 1:5000) were purchased from Abcam (Shanghai, China). Total RNA extraction, first-strand cDNA reverse transcription, polymerase chain reaction (PCR) kits and primers were obtained from TianGen Biotechnology Co., Ltd. (Beijing, China).

**2 Untargeted metabolomics analysis**

2.1 Sample Processing

First, collect brain tissue samples, weigh them, dilute with water (mass-to-volume ratio of 1:3), vortex for 3 minutes to prepare a suspension. For every 100μL of the suspension or serum, add 400μL of methanol (containing 12.5μg/mL of the internal standard 1,2-13C2 myristic acid), mix vigorously for 5 minutes, let it stand for 1 hour, then centrifuge at 14000×g for 10 minutes at 4°C. Pipette 100μL of the supernatant into a GC injection vial and use a Thermo Savant SPD2010-230 Speed Vac concentrator to evaporate the solvent under reduced pressure. Add 30μL of methoxamine pyridine solution (10mg/mL) to the dried powder, vortex vigorously for 2 minutes. Then, allow it to sit at room temperature for 16 hours for the oximation reaction. Subsequently, add 30μL of N-methyl-N-trimethylsilyl trifluoroacetamide (containing 1% trimethylchlorosilane) for trimethylsilylation and react for 1 hour. Finally, vortex the reaction mixture again for 30 seconds, add an external standard of methyl myristate heptane solution (30μg/mL), mix thoroughly, and use it for GC/MS detection.

2.2 GC-MS detection

The samples were detected using the Shimadzu GCMS-QP2010Ultra/SE gas chromatography-mass spectrometry system. In brief, 0.5μL of the sample was injected with a 1:10 split ratio (injection port temperature of 250°C) for chromatographic separation. The gas chromatography column used was an RTx-5MS fused silica capillary column, with helium as the carrier gas at a flow rate of 1.5 mL/min. The column temperature program was set as follows: initial temperature of 80°C for 3 minutes, followed by an increase of 20°C per minute until reaching 300°C, and held for 5 minutes. The ion source temperature was set at 200°C, and the transfer line temperature was set at 220°C. Ions were generated by an electron beam (energy -70eV), and the detection voltage was -950V. The mass spectrometry system used full scan acquisition (speed of 2500Hz), with a scanning range of 50-800 m/z and a solvent delay time of 300s. Chromatographic peaks were automatically detected, and GC-MS spectra were analyzed. Information such as mass spectra and retention times of chromatographic peaks was obtained. The detected compounds were identified by comparing them with the National Institute of Standards and Technology (NIST) library 2.0 (2008) spectral libraries (MAINLIB and PUBLIB) as well as Wiley 9 standard compound spectra (Wiley-VCH Verlag GmbH&Co.KgaA, Germany). Based on the comparison with standard compound mass spectra, characteristic quantitative ions and peak area quantitative data for the determined compounds were determined.

2.3 Data analysis

Based on the previously measured data, a data matrix was constructed with the measured samples as the first column, the retention time of chromatographic peaks as the first row, and the corresponding peak area data as the main body. The relative quantitative data of characteristic peaks (peak areas) were normalized and then imported into the SIMCA-P software (version 11.0) for multivariate statistical analysis. Principal Component Analysis (PCA) was used to observe the aggregation and dispersion of samples. Partial Least Squares-Discriminant Analysis (PLS-DA) was used to show the differences between groups. The PCA results were displayed as a scatter plot, representing the dispersion of samples, with closer dispersion indicating similar metabolic components. PLS-DA was used to identify groups. The t-test was performed on the differentially expressed metabolites identified by pattern recognition using SPSS software, and P<0.05 was considered statistically significant. Fold change (FC) analysis was performed on the metabolites that were statistically significant after the t-test. Heatmaps and clustering analysis of metabolites were analyzed using the OmicShare tool (http://www.omicshare.com/tools). After converting the metabolite names to Human Metabolite Database (HMDB) identifiers, MetaboAnalyst (http://www.metaboanalyst.ca/) was used to analyze the metabolic pathways of the differentially expressed metabolites.

**3 Primer sequence used in this study**

**Table S1** Primer sequence

| Gene | Sequence (5'->3') | |
| --- | --- | --- |
| *Adsl* | Forward primer | GAAAGCTACCGTTCTCCGCT |
|  | Reverse primer | GATGATGCCAGCAGCTTTCG |
| *Gad1* | Forward primer | CCAACCTGCGTCCTACAACA |
|  | Reverse primer | CATAAACAGTCGTGCCTGCG |
| *Actb* | Forward primer | CACCATGTACCCAGGCATTG |
|  | Reverse primer | CCTGCTTGCTGATCCACATC |

**4 Differences metabolites**

**Table S2** Differences metabolites of CIRI vs Sham.

| Name | Formula | Molecular Weight | RT [min] | m/z | FC | P value | VIP |
| --- | --- | --- | --- | --- | --- | --- | --- |
| Adenylosuccinic acid | C14 H18 N5 O11 P | 463.07 | 4.76 | 462.07 | 0.57 | 0.01 | 1.10 |
| Citric acid | C6 H8 O7 | 192.03 | 2.11 | 191.02 | 1.57 | 0.00 | 1.19 |
| Nicotinamide | C6 H6 N2 O | 122.05 | 1.97 | 123.06 | 0.51 | 0.00 | 1.03 |
| L-Glutamic acid | C5 H9 N O4 | 147.05 | 1.53 | 148.06 | 0.39 | 0.00 | 1.30 |
| Gamma-Aminobutyric acid | C4 H9 N O2 | 103.06 | 1.33 | 104.07 | 0.50 | 0.00 | 1.06 |
| Fumaric acid | C4 H4 O4 | 116.01 | 2.34 | 115.00 | 0.70 | 0.00 | 1.14 |
| L-Aspartic acid | C4 H7 N O4 | 133.04 | 1.30 | 132.03 | 0.45 | 0.00 | 1.32 |
| Docosahexaenoic acid | C22 H32 O2 | 328.24 | 9.70 | 327.23 | 4.10 | 0.00 | 1.41 |
| L-Canavanine | C5 H12 N4 O3 | 176.09 | 1.89 | 175.08 | 0.21 | 0.01 | 1.04 |
| Aflatoxin G1 | C17 H12 O7 | 350.03 | 4.71 | 351.04 | 0.34 | 0.00 | 1.43 |
| Maltotriose | C18 H32 O16 | 504.17 | 1.35 | 539.14 | 41.71 | 0.00 | 1.41 |
| N4-Acetylcytidine | C11 H15 N3 O6 | 285.10 | 4.84 | 284.09 | 5.18 | 0.00 | 1.49 |
| Lipoic acid | C8 H14 O2 S2 | 206.04 | 2.53 | 205.04 | 0.61 | 0.01 | 1.07 |
| Glutaric acid | C5 H8 O4 | 132.04 | 4.61 | 131.04 | 2.33 | 0.00 | 1.36 |
| trans-Cinnamic acid | C9 H8 O2 | 148.05 | 4.90 | 147.05 | 1.55 | 0.00 | 1.40 |
| Dulcitol | C6 H14 O6 | 182.08 | 1.29 | 217.05 | 3.25 | 0.00 | 1.42 |
| 12-Hydroxydodecanoic acid | C12 H24 O3 | 216.17 | 7.29 | 215.17 | 3.05 | 0.00 | 1.38 |
| L-lysine | C6 H14 N2 O2 | 146.11 | 1.23 | 145.10 | 2.03 | 0.00 | 1.27 |
| Prostaglandin B1 | C20 H32 O4 | 336.23 | 6.41 | 337.24 | 6.42 | 0.00 | 1.26 |
| Oleanolic acid | C30 H48 O3 | 478.34 | 9.80 | 479.35 | 4.09 | 0.00 | 1.28 |
| 10-Hydroxydecanoic acid | C10 H20 O3 | 188.14 | 6.60 | 187.13 | 7.26 | 0.00 | 1.47 |
| Palmitoylethanolamide | C18 H37 N O2 | 299.28 | 9.42 | 344.28 | 84.92 | 0.00 | 1.39 |
| D-Fructose 1,6-bisphosphate | C6 H14 O12 P2 | 340.00 | 2.03 | 322.99 | 6.85 | 0.00 | 1.27 |
| Phosphoethanolamine | C2 H8 N O4 P | 141.02 | 4.87 | 140.01 | 0.60 | 0.01 | 1.19 |
| Eicosapentaenoic acid | C20 H30 O2 | 302.22 | 9.30 | 301.22 | 2.42 | 0.00 | 1.35 |
| Pantothenic acid | C9 H17 N O5 | 219.11 | 5.00 | 218.10 | 1.97 | 0.00 | 1.29 |
| Senecionine | C18 H25 N O5 | 335.18 | 9.21 | 336.18 | 0.67 | 0.00 | 1.01 |
| Adipic acid | C6 H10 O4 | 146.06 | 5.19 | 145.05 | 1.90 | 0.00 | 1.25 |
| 11-Ketoetiocholanolone | C19 H28 O3 | 304.20 | 7.81 | 303.20 | 22.37 | 0.01 | 1.15 |
| Docosapentaenoic acid | C22 H34 O2 | 330.26 | 9.99 | 329.25 | 3.34 | 0.00 | 1.39 |
| Mupirocin | C26 H44 O9 | 500.29 | 7.42 | 523.28 | 2.57 | 0.01 | 1.11 |
| alpha-Farnesene | C15 H24 | 204.19 | 10.48 | 205.19 | 3.19 | 0.00 | 1.30 |
| Methionine | C5 H11 N O2 S | 149.05 | 1.98 | 150.06 | 0.45 | 0.00 | 1.08 |
| Theobromine | C7 H8 N4 O2 | 180.06 | 1.61 | 203.05 | 2.31 | 0.00 | 1.04 |
| Ursolic acid | C30 H48 O3 | 456.36 | 10.55 | 455.35 | 0.34 | 0.00 | 1.19 |
| 1-Methylhistidine | C7 H11 N3 O2 | 169.09 | 1.30 | 170.09 | 3.59 | 0.00 | 1.29 |
| isoleucine | C6 H13 N O2 | 131.09 | 3.03 | 132.10 | 2.26 | 0.00 | 1.28 |
| 4-Methylphenol | C7 H8 O | 108.06 | 5.50 | 107.05 | 3.87 | 0.00 | 1.34 |
| L-Pyroglutamic acid | C5 H7 N O3 | 129.04 | 2.08 | 130.05 | 0.62 | 0.00 | 1.10 |
| Dehydrocholic acid | C24 H34 O5 | 402.24 | 7.15 | 403.25 | 6.11 | 0.00 | 1.25 |
| Taurochenodeoxycholic acid | C26 H45 N O6 S | 499.30 | 6.52 | 498.29 | 4.91 | 0.00 | 1.22 |
| Lithocholic acid | C24 H40 O3 | 376.30 | 9.21 | 375.29 | 6.87 | 0.00 | 1.28 |
| Punicic Acid | C18 H30 O2 | 278.22 | 7.61 | 279.23 | 1.57 | 0.00 | 1.05 |
| Poncirin | C28 H34 O14 | 616.17 | 10.28 | 617.18 | 3.17 | 0.00 | 1.18 |
| Cannabidivarin | C19 H26 O2 | 286.20 | 7.09 | 304.23 | 18.89 | 0.00 | 1.52 |
| Anserine | C10 H16 N4 O3 | 240.12 | 1.29 | 241.13 | 0.34 | 0.00 | 1.22 |
| Palmitic acid | C16 H32 O2 | 256.24 | 10.23 | 255.23 | 1.61 | 0.00 | 1.11 |
| Elaidic acid | C18 H34 O2 | 282.26 | 8.17 | 281.25 | 2.67 | 0.00 | 1.02 |
| Adrenic acid | C22 H36 O2 | 332.27 | 10.46 | 331.26 | 5.54 | 0.00 | 1.37 |
| Valeric acid | C5 H10 O2 | 102.07 | 5.22 | 101.06 | 5.73 | 0.00 | 1.27 |
| 2-Furoic acid | C5 H4 O3 | 130.03 | 2.04 | 129.02 | 1.60 | 0.01 | 1.17 |
| Traumatic acid | C12 H20 O4 | 228.14 | 6.27 | 227.13 | 1.70 | 0.01 | 1.10 |
| Citicoline | C14 H26 N4 O11 P2 | 488.11 | 1.31 | 533.11 | 0.35 | 0.00 | 1.31 |
| Glu-Glu | C10 H16 N2 O7 | 276.10 | 1.57 | 275.09 | 0.38 | 0.00 | 1.30 |
| L-Isoleucine | C6 H13 N O2 | 131.09 | 3.32 | 132.10 | 1.77 | 0.01 | 1.19 |
| D-glutamine | C5 H10 N2 O3 | 146.07 | 1.55 | 147.08 | 0.41 | 0.00 | 1.23 |
| Deoxycholic acid | C24 H40 O4 | 392.29 | 7.08 | 391.29 | 3.12 | 0.01 | 1.09 |
| L-Ascorbate | C6 H8 O6 | 176.03 | 1.84 | 177.04 | 0.57 | 0.02 | 1.10 |
| Nonadecanoic acid | C19 H38 O2 | 298.29 | 11.29 | 297.28 | 1.80 | 0.03 | 1.03 |
| o-Toluic Acid | C8 H8 O2 | 136.05 | 2.00 | 137.06 | 0.06 | 0.03 | 1.04 |
| Cafestol | C20 H28 O3 | 316.20 | 6.13 | 317.21 | 4.33 | 0.03 | 1.06 |

**Table S3** Differences metabolites of H-FMN vs CIRI.

| Name | Formula | Molecular Weight | RT [min] | m/z | FC | Pvalue | VIP |
| --- | --- | --- | --- | --- | --- | --- | --- |
| Nicotinamide | C6 H6 N2 O | 122.05 | 1.97 | 123.06 | 2.61 | 0.00 | 1.55 |
| L-Glutamic acid | C5 H9 N O4 | 147.05 | 1.53 | 148.06 | 2.34 | 0.00 | 1.24 |
| Gamma-Aminobutyric acid | C4 H9 N O2 | 103.06 | 1.33 | 104.07 | 2.22 | 0.00 | 1.33 |
| Fumaric acid | C4 H4 O4 | 116.01 | 2.34 | 115.00 | 1.54 | 0.00 | 1.41 |
| L-Aspartic acid | C4 H7 N O4 | 133.04 | 1.30 | 132.03 | 2.23 | 0.00 | 1.39 |
| Docosahexaenoic acid | C22 H32 O2 | 328.24 | 9.70 | 327.23 | 0.30 | 0.00 | 1.25 |
| L-Canavanine | C5 H12 N4 O3 | 176.09 | 1.89 | 175.08 | 4.35 | 0.00 | 1.10 |
| Aflatoxin G1 | C17 H12 O7 | 350.03 | 4.71 | 351.04 | 2.23 | 0.00 | 1.08 |
| Maltotriose | C18 H32 O16 | 504.17 | 1.35 | 539.14 | 0.04 | 0.00 | 1.40 |
| Lipoic acid | C8 H14 O2 S2 | 206.04 | 2.53 | 205.04 | 1.72 | 0.01 | 1.23 |
| Glutaric acid | C5 H8 O4 | 132.04 | 4.61 | 131.04 | 0.53 | 0.00 | 1.07 |
| Dulcitol | C6 H14 O6 | 182.08 | 1.29 | 217.05 | 0.37 | 0.00 | 1.25 |
| 12-Hydroxydodecanoic acid | C12 H24 O3 | 216.17 | 7.29 | 215.17 | 0.44 | 0.00 | 1.06 |
| L-lysine | C6 H14 N2 O2 | 146.11 | 1.23 | 145.10 | 0.57 | 0.00 | 1.07 |
| Prostaglandin B1 | C20 H32 O4 | 336.23 | 6.41 | 337.24 | 0.23 | 0.02 | 1.01 |
| Oleanolic acid | C30 H48 O3 | 478.34 | 9.80 | 479.35 | 0.26 | 0.00 | 1.30 |
| 10-Hydroxydecanoic acid | C10 H20 O3 | 188.14 | 6.60 | 187.13 | 0.22 | 0.00 | 1.22 |
| Palmitoylethanolamide | C18 H37 N O2 | 299.28 | 9.42 | 344.28 | 0.02 | 0.00 | 1.42 |
| Phosphoethanolamine | C2 H8 N O4 P | 141.02 | 4.87 | 140.01 | 1.53 | 0.01 | 1.07 |
| Adipic acid | C6 H10 O4 | 146.06 | 5.19 | 145.05 | 0.51 | 0.00 | 1.38 |
| 11-Ketoetiocholanolone | C19 H28 O3 | 304.20 | 7.81 | 303.20 | 0.04 | 0.01 | 1.22 |
| Mupirocin | C26 H44 O9 | 500.29 | 7.42 | 523.28 | 0.36 | 0.00 | 1.29 |
| Xylitol | C5 H12 O5 | 152.07 | 1.30 | 151.06 | 2.26 | 0.00 | 1.28 |
| alpha-Farnesene | C15 H24 | 204.19 | 10.48 | 205.19 | 0.30 | 0.00 | 1.40 |
| N-Oleoyl dopamine | C26 H43 N O3 | 417.32 | 10.27 | 400.32 | 0.24 | 0.00 | 1.49 |
| Arachidonic acid | C20 H32 O2 | 304.24 | 9.78 | 303.23 | 1.57 | 0.00 | 1.27 |
| Methionine | C5 H11 N O2 S | 149.05 | 1.98 | 150.06 | 2.72 | 0.00 | 1.38 |
| Theobromine | C7 H8 N4 O2 | 180.06 | 1.61 | 203.05 | 0.43 | 0.00 | 1.10 |
| 4-Methylphenol | C7 H8 O | 108.06 | 5.50 | 107.05 | 0.31 | 0.00 | 1.24 |
| D-Xylulose 5-phosphate | C5 H11 O8 P | 230.02 | 1.54 | 229.01 | 1.75 | 0.00 | 1.31 |
| Phenylacetylglycine | C10 H11 N O3 | 193.07 | 5.44 | 192.07 | 0.31 | 0.00 | 1.21 |
| Arachidic acid | C20 H40 O2 | 312.30 | 11.32 | 311.30 | 0.30 | 0.00 | 1.22 |
| Sulfoacetic acid | C2 H4 O5 S | 139.98 | 1.97 | 138.97 | 2.92 | 0.00 | 1.29 |
| L-Pyroglutamic acid | C5 H7 N O3 | 129.04 | 2.08 | 130.05 | 1.59 | 0.00 | 1.16 |
| Dehydrocholic acid | C24 H34 O5 | 402.24 | 7.15 | 403.25 | 0.18 | 0.00 | 1.26 |
| Lithocholic acid | C24 H40 O3 | 376.30 | 9.21 | 375.29 | 0.18 | 0.00 | 1.22 |
| Cannabidivarin | C19 H26 O2 | 286.20 | 7.09 | 304.23 | 0.15 | 0.00 | 1.10 |
| Kynurenic acid | C10 H7 N O3 | 189.04 | 5.29 | 188.04 | 0.39 | 0.00 | 1.11 |
| Palmitic acid | C16 H32 O2 | 256.24 | 10.23 | 255.23 | 0.61 | 0.00 | 1.25 |
| Elaidic acid | C18 H34 O2 | 282.26 | 8.17 | 281.25 | 0.30 | 0.00 | 1.42 |
| Stearic acid | C18 H36 O2 | 284.27 | 11.08 | 283.26 | 0.54 | 0.00 | 1.30 |
| Glycyl-L-leucine | C8 H16 N2 O3 | 188.12 | 5.13 | 189.12 | 2.75 | 0.00 | 1.17 |
| Adrenic acid | C22 H36 O2 | 332.27 | 10.46 | 331.26 | 0.20 | 0.00 | 1.33 |
| S-Adenosylhomocysteine | C14 H20 N6 O5 S | 384.12 | 2.29 | 385.13 | 1.61 | 0.00 | 1.08 |
| Sphinganine | C18 H39 N O2 | 301.30 | 6.46 | 302.31 | 0.49 | 0.01 | 1.07 |
| Valeric acid | C5 H10 O2 | 102.07 | 5.22 | 101.06 | 0.15 | 0.00 | 1.46 |
| Phenylacetaldehyde | C8 H8 O | 120.06 | 5.15 | 119.05 | 0.65 | 0.01 | 1.20 |
| Cannabidiolic acid | C22 H30 O4 | 358.21 | 10.05 | 357.21 | 0.73 | 0.01 | 1.16 |
| Citicoline | C14 H26 N4 O11 P2 | 488.11 | 1.31 | 533.11 | 2.47 | 0.00 | 1.21 |
| Glu-Glu | C10 H16 N2 O7 | 276.10 | 1.57 | 275.09 | 2.03 | 0.00 | 1.05 |
| D-glutamine | C5 H10 N2 O3 | 146.07 | 1.55 | 147.08 | 2.38 | 0.00 | 1.21 |
| Methyltestosterone | C20 H30 O2 | 302.22 | 9.63 | 303.23 | 0.65 | 0.02 | 1.13 |
| D-Gluconic acid | C6 H12 O7 | 196.06 | 1.30 | 195.05 | 0.53 | 0.02 | 1.01 |
| L-Palmitoylcarnitine | C23 H45 N O4 | 399.33 | 10.45 | 422.32 | 0.45 | 0.02 | 1.08 |
| O-Phosphorylethanolamine | C2 H8 N O4 P | 141.02 | 1.22 | 140.01 | 1.89 | 0.02 | 1.14 |
| Stearamide | C18 H37 N O | 283.29 | 10.37 | 284.29 | 0.62 | 0.03 | 1.03 |
| Nonadecanoic acid | C19 H38 O2 | 298.29 | 11.29 | 297.28 | 0.57 | 0.03 | 1.05 |
| 3-hydroxy-3-methylpentanedioic acid | C6 H10 O5 | 184.03 | 1.34 | 185.04 | 0.28 | 0.04 | 1.03 |
| Homoarginine | C7 H16 N4 O2 | 188.13 | 1.30 | 189.13 | 0.57 | 0.04 | 1.13 |
